# Regulation of lipid droplets accumulation by the Hippo–YAP/COX2 signaling pathway in neomycin-induced ototoxicity

**DOI:** 10.1038/s41420-026-03115-w

**Published:** 2026-04-16

**Authors:** Wenli Hao, Song Gao, Suhan Guo, Jingjing Luo, Siyu Li, Junze Lu, Lulu Jiang, Jie Lu, Nan Wang, Aijia Ran, Xiaoyun Qian, Xia Gao, Chenjie Yu, Cheng Cheng

**Affiliations:** 1 Department of Otolaryngology-Head and Neck Surgery, Jiangsu Provincial Key Medical Discipline (Laboratory), Nanjing Drum Tower Hospital, Affiliated Hospital of Medical School, Nanjing University, Nanjing, China; 2 Jiangsu Province High-Tech Key Laboratory for Bio-Medical Research, Southeast University, Nanjing, China; 3 Northern Jiangsu People’s Hospital Affiliated to Yangzhou University, Yangzhou, China; 4 Research Institute of Otolaryngology, Nanjing, China; 5 Department of Otolaryngology-Head and Neck Surgery, Nanjing Drum Tower Hospital, Affiliated Hospital of Medical School, MOE Key Laboratory of Model Animal for Disease Study, Nanjing University, Nanjing, China

**Keywords:** Lipid signalling, Gene regulation

## Abstract

Lipid metabolism is an important biological process that maintains the dynamic balance of several key functions, such as intracellular energy metabolism, signal transduction, and membrane remodeling. However, the role of lipid metabolism in auditory function and the underlying mechanisms remain unclear. Our results reveal that the neomycin exposure disrupts lipid metabolism in auditory system. We find that neomycin-induced hair cells (HC) damage leads to abnormal lipid droplets (LD) accumulation. Further research reveals that decreased YAP expression is a key factor that contributes to abnormal LD accumulation. In both in vivo and in vitro studies, *Yap* overexpression reduces abnormal LD accumulation and mitigated HC damage. To further investigate its downstream mechanisms, we perform a cross-analysis of *Yap*-related and lipid metabolism–related genes, identifying that *Cox2* is a key downstream target of *Yap* that contributes to LD accumulation and HC damage. Our work provides clear evidence for the role of lipid metabolism in neomycin-induced hearing loss and elucidates underlying mechanism of *Yap/Cox2* pathway. These findings provide new perspectives and avenues for the clinical treatment of sensorineural hearing loss.

## Introduction

According to the World Health Organization (WHO), approximately 1.5 billion people worldwide have varying degrees of hearing loss, with 34 million of them being children. It is projected that the number of people with hearing loss will increase to 9 billion by 2050 [1, 2]. Aminoglycoside antibiotics such as neomycin and kanamycin are critical therapeutic agents for the treatment of severe infections and Meniere’s disease. However, their clinical utility is limited because of their potential to cause irreversible sensorineural hearing loss (SNHL) [3, 4]. Clinical studies indicate that 20-47% of patients treated with aminoglycosides develop hearing loss owing to ototoxic side effects. Hearing loss caused by aminoglycoside substances is typically dose-dependent and cumulative. In addition, the sensitivity of hair cells (HC) to aminoglycosides differs across various frequency ranges. Initially, aminoglycosides primarily cause high-frequency hearing loss before progressively causing low-frequency hearing loss [5, 6]. Permanent hearing loss caused by such ototoxic drugs is mainly because of the non-renewability of HC [7]. Therefore, the investigation of biological mechanisms through which neomycin induces HC death and finding ways to repair this neomycin-induced damage are crucial for treating SNHL.

Abnormal lipid metabolism is widely discussed in the context of sensorineural deafness. With increasing age, cardiovascular risk factors such as triglycerides, serum total cholesterol, low-density lipoprotein, and high-density lipoprotein increase and are associated with hearing loss [8]. The use of statin drugs, a class of drugs used to lower cholesterol levels, can improve inner ear microcirculation by reducing lipoprotein concentration, thereby protecting hearing [9]. In the doxorubicin-induced aging state, HEI-OC1 cells showed increased cholesterol levels, lysosomal damage, and increased autophagy. Increasing cholesterol supply can exacerbate lysosomal damage and autophagosome accumulation, whereas low-cholesterol treatment reduces the accumulation of lysosomes and autophagosomes [10]. In an aging mouse model, direct delivery of 3-hydroxy-3-methylglutaryl coenzyme A (HMG-CoA) reductase inhibitors, also known as statin drugs, into the cochlea through artificial ear canal surgery can inhibit noise-induced HC loss and hearing loss [11]. These researches indicated that lipid alterations are related to auditory impairment. However, the role and mechanism of lipid metabolism in ototoxic drug-induced hearing loss remain further in-depth investigation.

The Hippo/YAP pathway is a conserved kinase cascade that regulates various cellular processes, including cell survival, proliferation, and migration [12]. Various stimuli, including cell–cell adhesion, mechanical stiffness of the extracellular matrix, and drug stimulation, activate the Hippo/YAP signaling pathway [13]. In the cochlear tissue, the Hippo/YAP signaling pathway is believed to be involved in organ development and damage. Activation of the Hippo signaling pathway leads to the degradation of *SKP2* by inhibiting YAP activation, thereby causing HC to exit the cell cycle during development [14]. YAP overexpression can induce HC regeneration [15]. In vitro experiments have shown that the Hippo/YAP signaling pathway can reduce aminoglycoside drug–induced HC damage [16]. Cisplatin-induced HC injury suppresses YAP expression, whereas pharmacological YAP activation (via LAT1-IN-1) reduces oxidative stress and ferroptosis by upregulating ferritin light chain (FTL) and transferrin receptor (TFRC). Conversely, YAP inhibition (verteporfin) exacerbates the damage, confirming its protective role [17]. Recent studies have also shown a connection between YAP and lipid metabolism. In macrophages, YAP activation reduces PLIN2 expression and decreases LD accumulation. YAP knockout exacerbates high-fat diet–induced lipid accumulation [18]. YAP has been reported to be involved in the reprogramming of lipid metabolism in cancer cells [19, 20].

Cyclooxygenase 2 (COX2), encoded by *Ptgs2*, catalyzes prostaglandin synthesis (e.g., PGE₂) from arachidonic acid [21]. As a joint enzyme in prostaglandin biosynthesis, COX2 affects apoptosis and survival of tumor cells through multiple mechanisms. For example, COX2 can promote the survival of tumor cells by activating the PI3K–AKT–PPARγ pathway [22, 23]. YAP directly transcriptionally regulates *PTGS2*, as demonstrated in *NF2*-null Schwann cells, where YAP knockdown reduced COX2 expression [24]. Although not explicitly studied in cochlear lipid metabolism, COX2 is a lipid metabolism mediator, and its YAP-dependent expression suggests a potential otoprotective axis [25, 26]. In vestibular schwannomas, *PTGS2* correlates with YAP targets (*CYR61* and *CTGF*), implicating this pathway in sensory cell survival.

In this study, we verified that the pharmacological activation of YAP could rescue neomycin-induced hearing loss and HC loss and explored the downstream targets and mechanisms of possible effects on YAP. Our results indicate that YAP regulates lipid metabolism homeostasis by regulating the transcriptional expression of COX2, thereby affecting the survival of HC.

## Results

### Neomycin causes abnormal lipid metabolism

**Fig. 1:**
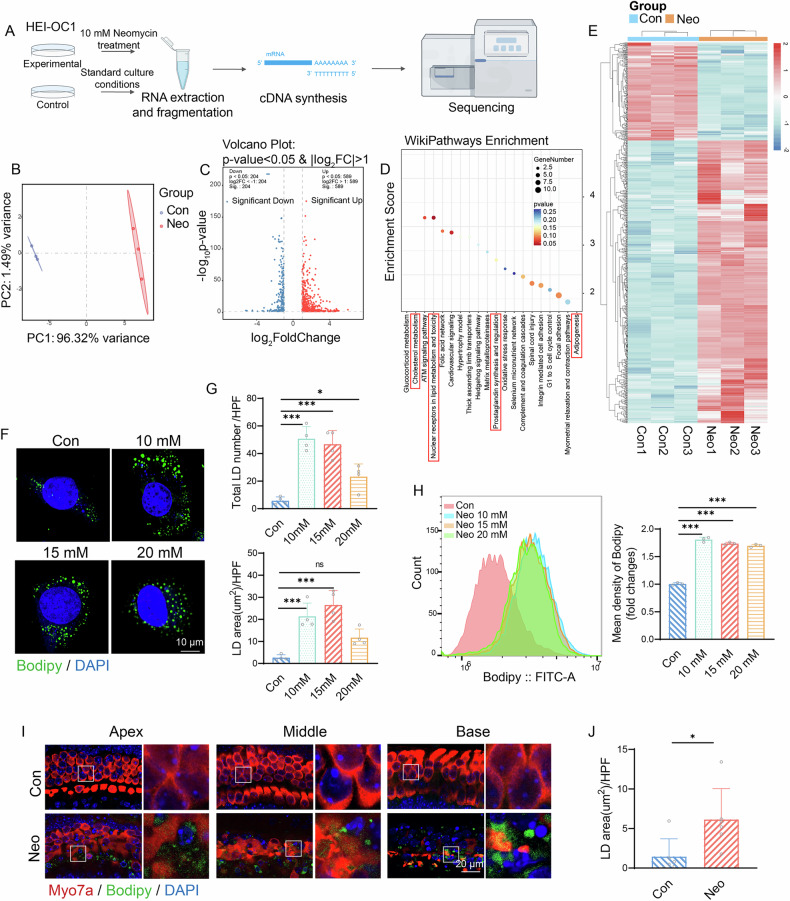
Neomycin causes abnormal lipid metabolism. **A** Protocol of cell transcriptome sequencing. **B** Principal Component Analysis (PCA) showing the different groups. **C** Volcano plots showing the differentially expressed genes between control group and neomycin-treated group. **D** Wikipathways Enrichment shows the top 20 different metabolic pathways. **E** Cluster heatmap showing the clustering of differentially expressed genes between the control group and the neomycin treatment group. **F** Bodipy (green) and DAPI (blue) staining of HEI-OC1 cells treated with different concentrations (0 mM, 10 mM, 15 mM, 20 mM) of neomycin. Scale bar = 10 μm. **G** Statistical analysis of the area and quantity of LD in (**F**). Data are represented as means ± SD. Data from multiple groups was analyzed by one-way ANOVA. *n* = 4. **H** Flow cytometric analysis of the immunofluorescence intensity of bodipy. Data are represented as means ± SD. Data from multiple groups was analyzed by one-way ANOVA. *n* = 3. **I** Myosin7a (red), bodipy (green) and DAPI (blue) staining of the cultured cochlear explant. Scale bar = 20 μm. **J** Statistical analysis of the area of LD in (**I**). Data are represented as means ± SD and analyzed by the Student’s t-test. *n* = 6. *p < 0.05, **p < 0.01, and ***p < 0.001.

To explore the specific mechanism underlying neomycin-induced hearing loss, we conducted transcriptome sequencing of control and neomycin-treated HEI-OC1 cells (Fig. 1A). Principal component analysis (PCA) revealed a clear distinction between the control and neomycin-treated groups (Fig. 1B). In the neomycin-treated group, 589 genes were upregulated and 204 genes were downregulated relative to the control group (Fig. 1C, E). In the subsequent pathway analysis, Wiki pathways enrichment analysis of metabolic-related pathways revealed several lipid metabolism pathways among the top 20 pathways (indicated by the red box) (Fig. 1D). The relevant pathways include Cholesterol metabolism, Nuclear receptors in lipid metabolism and toxicity, Prostaglandin synthesis and regulation, and Adipogenesis. The changes in cholesterol metabolism mainly manifest as increased synthesis and uptake, decreased esterification and excretion, indicating excessive free cholesterol [27]. The imbalance in nuclear receptors in lipid metabolism and toxicity indicates lipid overload and the accumulation of toxic intermediate products. The imbalance in prostaglandin synthesis and regulation indicates the occurrence of acute inflammation [28]. Moreover, the increase in Adipogenesis also confirms the accumulation of lipid droplets [29]. LD, which are essential organelles for the storage of cholesterol and triglycerides (TG), play a significant role in lipid metabolism. HEI-OC1 cells were treated with increasing concentrations of neomycin (10, 15, and 20 mM) and then stained with bodipy to analyze both the number and area of LD. The results showed that neomycin induced abnormal LD accumulation, with the greatest number of LD observed at 10 mM concentration (Fig. 1F, G). The accumulation of lipid droplets initially increases and then decreases as the injury intensifies, reflecting the cell’s changing ability to regulate lipid toxicity in response to injury stimuli. Under mild injury conditions, to prevent the harmful accumulation of free, saturated, long-chain fatty acids in the cytoplasm, intracellular lipid accumulation exceeds the normal metabolic capacity as the severity of the injury increases. Excessive saturated long-chain fatty acids can trigger significant endoplasmic reticulum stress and mitochondrial damage, leading to proteolytic cleavage of proteins on the surface of lipid droplets and structural damage to lipid droplets, thereby reducing their abundance [30]. Flow cytometry analysis also revealed consistent results, showing a significantly increase in bodipy immunofluorescence intensity upon neomycin treatment (Fig. 1H). We also applied neomycin treatment in cochlear explants and found that LD accumulated in the HC of the apical, middle, and basal turns of the cochlea (Fig. 1I, J). These results confirmed that neomycin disrupts lipid metabolism in HC.

### Neomycin injury leads to activation of Hippo signaling pathways and suppression of YAP expression in HC

**Fig. 2:**
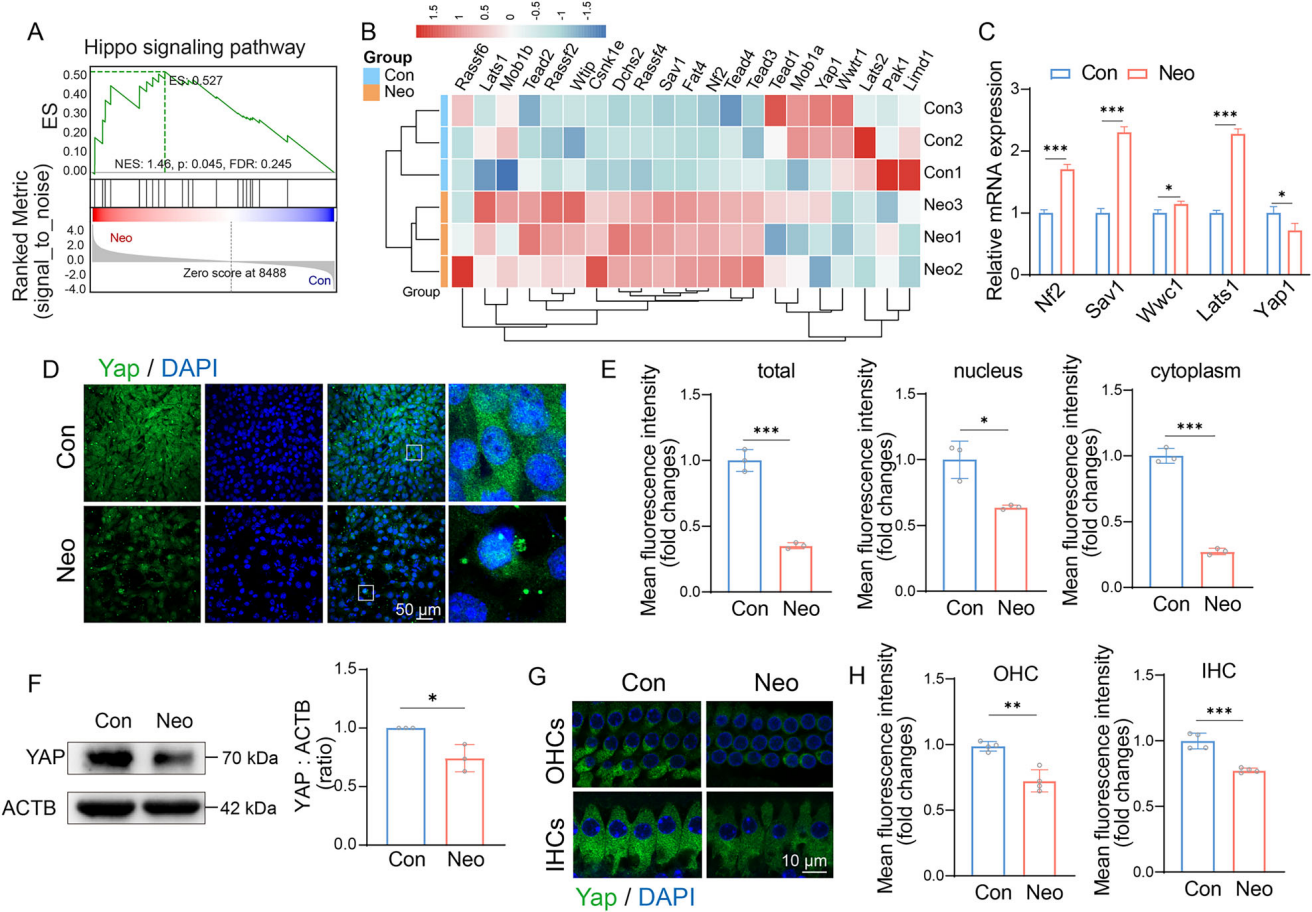
Neomycin injury leads to activation of Hippo/Yap signaling pathways in HC. **A** GSEA for Hippo signaling pathway. **B** Heatmap of key genes in Hippo signaling pathway. **C** RT-qPCR analysis for the expression levels of *Nf2*, *Sav1*, *Wwc1*, *Lats1*, *Yap1* in control and neomycin-treated HEI-OC1 cells. **D** Co-staining images of Yap (green) and DAPI (blue) in control and neomycin-treated HEI-OC1 cells. Scale bar = 50 μm. **E** Statistical analysis of the average fluorescence intensity of YAP in the cytoplasm and nucleus in control and neomycin-treated HEI-OC1 cells. Data are represented as means ± SD and analyzed by the Student’s t-test. *n* = 3. **F** Western blot of YAP expression in control and treatment of 24 h neomycin. ACTB was used as an internal reference. *n* = 3. **G** Confocal images of the organ of Corti immunolabeled for Yap (green), nuclei were stained blue by DAPI. Scale bar = 10 μm. **H** Statistical analysis of the average fluorescence intensity of YAP in OHC and IHC in (**G**). OHC: outer hair cell, IHC: inner hair cell, *n* = 4–6. Data are represented as means ± SD and analyzed by Student’s t-test. *p < 0.05, **p < 0.01, and ***p < 0.001.

In previous studies, we discovered that inhibiting the Hippo/YAP signaling pathway could protect HC from neomycin-induced damage in vitro [16]. Meanwhile, YAP is reported to regulate lipid homeostasis via transcriptional control of *SREBP1*, *FASN*, and *PPARγ* [18, 19, 31]. Therefore, we investigated whether the Hippo/YAP pathway is involved in the abnormal accumulation of LD induced by neomycin. Using sequencing data, we conducted Gene Set Enrichment Analyses (GSEA) to examine alterations in the Hippo/YAP signaling pathway during neomycin-induced damage. The results indicated that the Hippo/YAP signaling pathway was activated (Fig. 2A), and the cluster heatmap showed that 13 Hippo/YAP signaling related genes were upregulated and 11 genes were downregulated (Fig. 2B). We also performed quantitative PCR (qPCR) to validate the RNA sequencing results and found that the transcription levels of key genes in the Hippo signaling pathway, including *Nf2*, *Sav1*, *Wwc1*, and *Lats1*, were increased. In contrast, the transcriptional level of *Yap* was decreased (Fig. 2C). Immunofluorescence staining showed that YAP expression in both nucleus and cytoplasm of HEI-OC1 cells distinctly decreased after 24 h of treatment with 10 mM neomycin (Fig. 2D, E). Western blotting also showed that after neomycin treatment, the expression of YAP in HEI-OC1 cells was decreased (Fig. 2F). Neomycin was subcutaneously injected into postnatal (P) 8 C57BL/6 mice for 7 days, and the auditory brainstem response (ABR) was tested to detect the hearing of the mice at P30. ABR thresholds were significantly increased in mice that received neomycin compared with controls (Supplementary Fig. 1A). The basilar membranes were stained with an anti-YAP antibody, and quantitative analysis demonstrated that the mean density of YAP was reduced in the neomycin group compared to that in the control group (Fig. 2G, H). Owing to the immortalization modification of HEI - OC1 cells, the expression of YAP was also observed in the cell nuclei of this cell line. Our data confirmed that neomycin treatment increases YAP degradation, indicating that the Hippo/YAP signaling pathway plays an essential regulatory role in neomycin-induced HC damage.

### YAP inhibition drives neomycin-induced abnormal accumulation of LD and HC damage

**Fig. 3:**
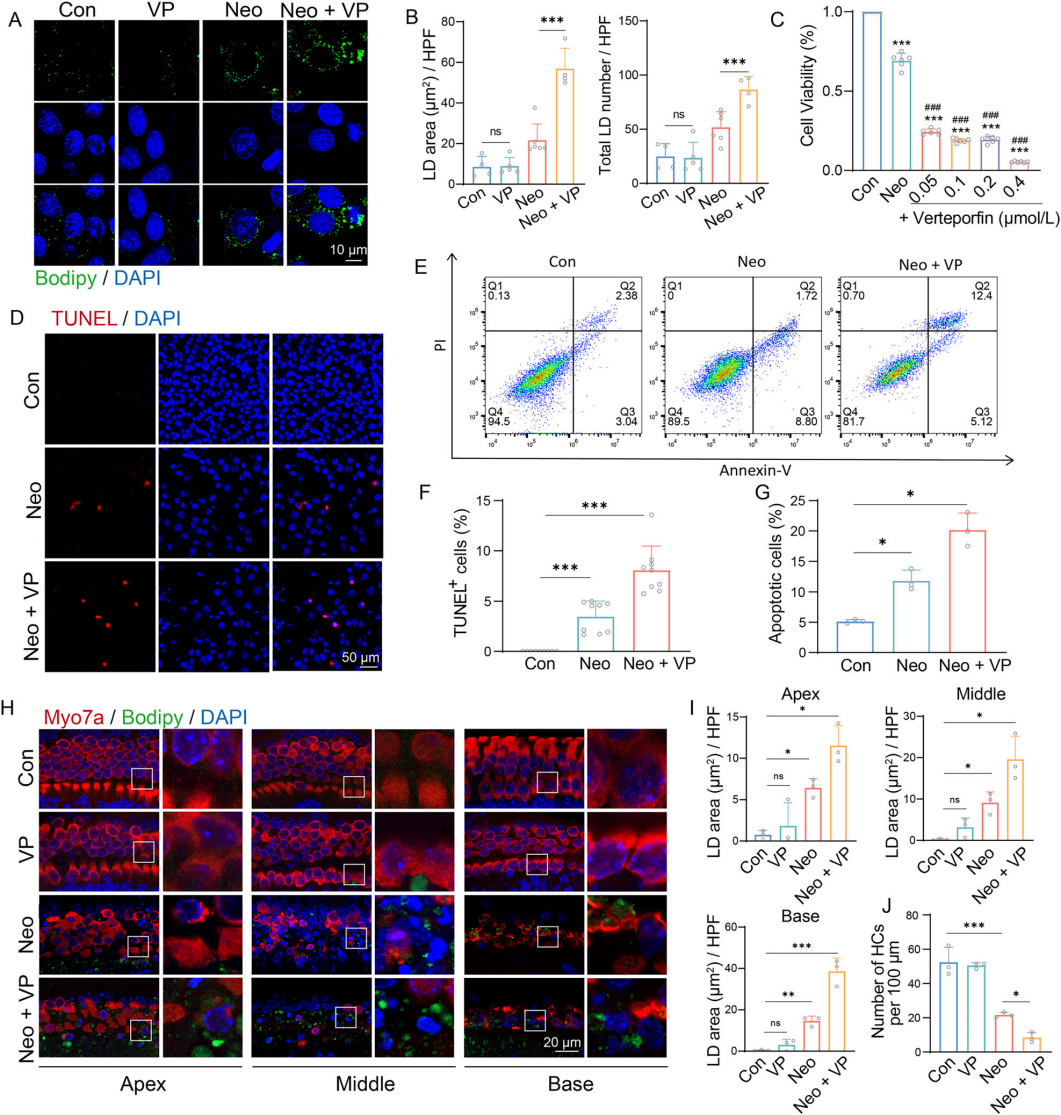
The inhibition of Yap promotes the neomycin-induced abnormal accumulation of LD and HC damage. **A** Bodipy (green) and DAPI (blue) staining of HEI-OC1 cells in the control (Con), verteporfin-only (VP), neomycin-only (Neo) and neomycin + verteporfin (Neo + VP) groups. Scale bars = 10 μm. **B** Statistical analysis was conducted on the area and quantity of LD in each high-power field (HFP) of (**A**). *n* = 4. Data are represented as means ± SD. Data from multiple groups was compared by the one-way ANOVA. **C** Cell viability of HEI-OC1 cells treated with neomycin and different concentrations of verteporfin for 24 h, measured by CCK-8 assay. *n* = 6. Data are represented as means ± SD. Data from multiple groups was compared by the one-way ANOVA. * represents the comparison with control group; # represents the comparison with the neomycin group. **D** TUNEL (red) and DAPI (blue) double staining showing the proportion of apoptotic HEI-OC1 cells after different treatments. **E** The percent of apoptotic cells after 24 h of neomycin and verteporfin treatment was measured by flow cytometry. **F** Statistical analysis of the proportion of TUNEL+ cells in (**D**). *n* = 9. Data are represented as means ± SD. Data from multiple groups was compared by the one-way ANOVA. **G** Statistical analysis of the proportion of apoptotic cells in (**E**). *n* = 3. Data are represented as means ± SD. Data from multiple groups was compared by the one-way ANOVA. **H** Myosin7a (red), bodipy (green) and DAPI (blue) staining of cochlear explants in the control, verteporfin-only, neomycin-only and neomycin + verteporfin groups. Scale bars = 20 μm. **I** Statistical analysis of the area of LD in HFP of the apical, middle, basal turns in the four groups. *n* = 3. **J** Statistical analysis of the number of HC in the middle turns of the cultured cochlear explant in the four groups. *n* = 3. Data are represented as means ± SD. Data from multiple groups was compared by the one-way ANOVA. ns = not significant, *p < 0.05, **p < 0.01, and ***p < 0.001, ^#^*p* < 0.05, ^##^*p* < 0.01, and ^###^*p* < 0.001.

Verteporfin inhibits YAP function by preventing YAP-TEAD binding [32]. We first examined its safe concentration for HEI-OC1 cells using a CCK8 assay. HEI-OC1 cells were exposed to increasing concentrations of verteporfin (0.05, 0.1, 0.2, 0.4, and 0.8 μM) for 24 h. No significant cytotoxicity was observed at concentrations below 0.4 μM (Supplementary Fig. 1B). Therefore, 0.4 μM was selected as the non-toxic concentration for subsequent experiments. To examine the role of YAP in regulating lipid metabolism during neomycin-induced ototoxicity, we examined the accumulation of LD using bodipy staining. Compared to cells treated with neomycin alone, co-treatment with verteporfin and neomycin significantly increased LD accumulation in HEI-OC1 cells (Fig. 3A, B). To further elucidate the functional impact of verteporfin on neomycin-induced cellular injury, HEI-OC1 cells were exposed to 10 mM neomycin in combination with varying concentrations of verteporfin (0.05, 0.1, 0.2, and 0.4 μM). The findings demonstrate that verteporfin exacerbated neomycin-induced cytotoxicity in HEI-OC1 cells in a dose-dependent manner, with increasing verteporfin concentrations correlating with progressively intensified cellular damage (Fig. 3C). We also evaluated the ability of verteporfin in inhibiting YAP activity by analyzing the downstream target genes of YAP under neomycin-induced damage. The transcription levels of *Birc5* and *Ccn2* were decreased (Supplementary Fig. 1C). Apoptotic HEI-OC1 cells were marked by TUNEL and cleaved-caspase3. Compared to the neomycin-treated group, the number of apoptotic cells increased in the group treated with both verteporfin and neomycin (Fig. 3D, F, Supplementary Fig. 1E, F). Western blot analysis showed cleaved-caspase3 expression was increased after verteporfin treatment (Supplementary Fig. 1D). Flow cytometric analysis was conducted utilizing propidium iodide (PI) to label non-viable cells and Annexin V to identify apoptotic cells. The proportion of apoptotic HEI-OC1 cells in the group co-treated with 0.4 μM verteporfin and 10 mM neomycin was significantly greater compared to the group treated with neomycin alone (Fig. 3E, G).

We validated our findings using cultured cochlear explants and found that treatment with verteporfin alone did not induce abnormal LD accumulation in HC or cause HC loss. However, when combined with neomycin, verteporfin exacerbated the abnormal accumulation of LD in HC, with the increased HC death (Fig. 3H–J). Taken together, verteporfin treatment increased abnormal LD accumulation induced by neomycin in both HEI-OC1 cells and cultured cochlear explants, which was accompanied by a corresponding increase in cell apoptosis.

### YAP inhibition exacerbated neomycin-induced hearing loss and HC damage

**Fig. 4:**
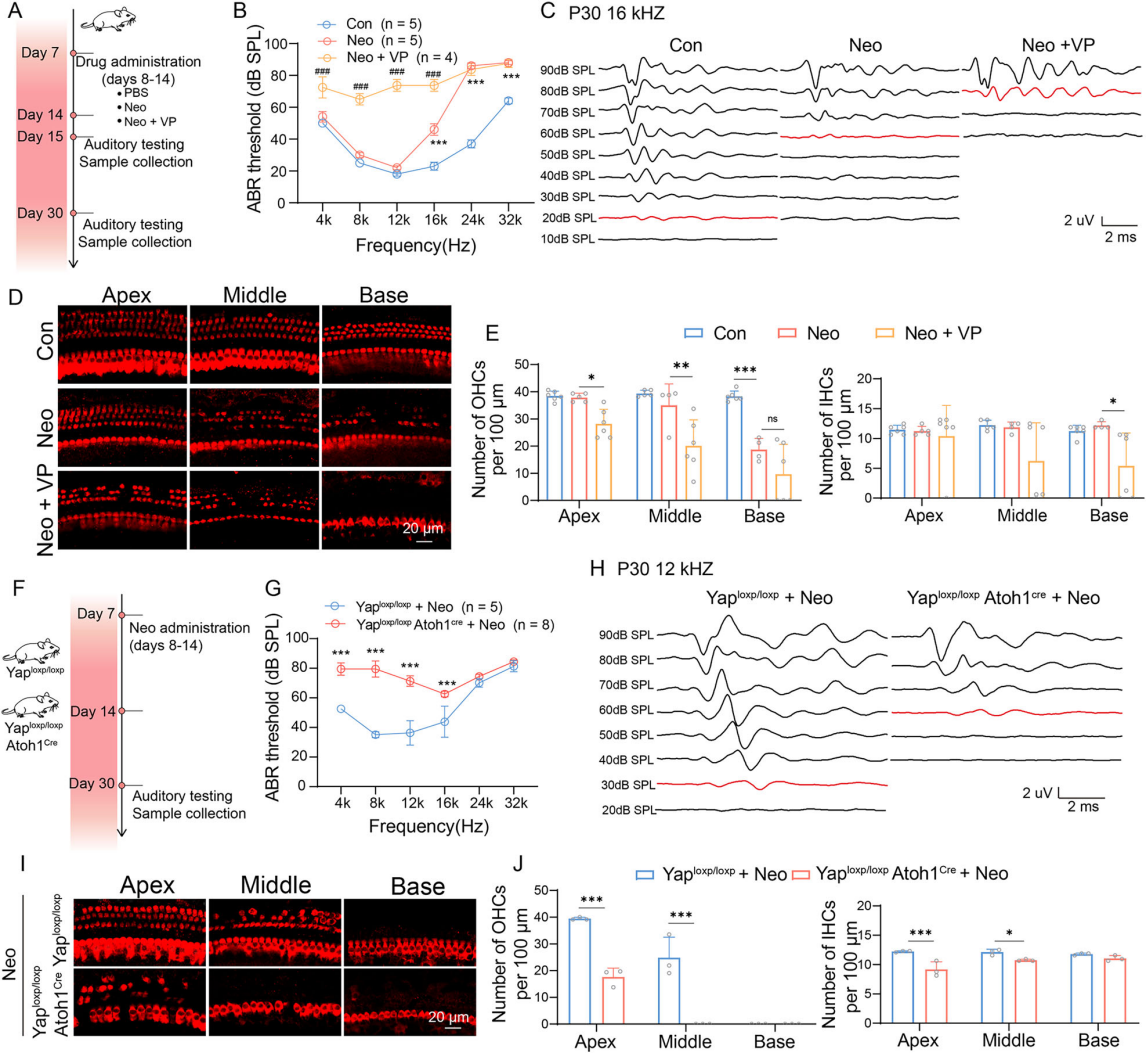
Downregulation of Yap exacerbate neomycin induced hearing loss. **A** Schematic illustration the experiments of neomycin and verteporfin administration into C57 BL/6 mice. **B** ABR thresholds in response to tone-pip stimuli (4, 8, 12, 16, 24, and 32 kHz) in C57 BL/6 mice with treatment of neomycin injection or neomycin and verteporfin injection. Data are represented as means ± SEM. Data from multiple groups was compared by the two-way ANOVA. **C** ABR waveforms at 16 kHz across all sound pressure levels; the red line indicated the ABR thresholds. **D** Immunostaining with myosin7a in apical, middle, and basal turns of cochleae from different groups. Scale bars = 20 μm. **E** Statistical analysis of number of HC in (**D**). *n* = 4–6. Data are represented as means ± SD. Data from multiple groups was compared by the two-way ANOVA. **F** Schematic illustration of the experiments involving neomycin administration in cKO mice. **G** ABR thresholds in response to tone-pip stimuli (4, 8, 12, 16, 24, and 32 kHz) in cKO mice. Data are represented as means ± SEM. Data from multiple groups was compared by the two-way ANOVA. **H** ABR waveforms at 12 kHz across all sound pressure levels; the red line indicated the ABR thresholds. **I** Immunostaining with myosin7a (red) in apical, middle, and basal turns of cochleae from different groups. Scale bars = 20 μm. **J** Statistical analysis of number of OHC and IHC in (**I**). *n* = 3. Data are represented as means ± SD. p value were analyzed by the unpaired two-tailed t-test. *p < 0.05, **p < 0.01, and ***p < 0.001.

To assess the ototoxic potential of verteporfin, mice were injected with verteporfin (1 mg/kg) from P8 to P14. Verteporfin did not induce hearing loss or HC damage at P30 (Supplementary Fig. 2A–C). We then injected 100 mg/kg neomycin or 1 mg/kg verteporfin into wild-type mice for 7 consecutive days from P8 to P14, and the ABR threshold and HC survival in neomycin + verteporfin injected mice were compared with neomycin-injected mice at P15 and P30 (Fig. 4A). The transcriptional levels of YAP downstream target genes (*Birc5, Ccn2*, and *Nuak2*) in the cochlea were reduced after verteporfin treatment, confirming the inhibitory effect of verteporfin on YAP function (Supplementary Fig. 2D). The ABR results showed that neomycin treatment significantly increased hearing thresholds at 24 and 32 kHz in P15 mice. Notably, co-administration of verteporfin further increased the ABR thresholds compared to treatment with neomycin alone (Supplementary Fig. 2E). By P30, mice treated with both neomycin and verteporfin exhibited an even greater deterioration in auditory thresholds (Fig. 4B, C). The immunofluorescence staining results showed that verteporfin exacerbated neomycin-induced HC damage at P15, with increased HC damage observed at P30 (Supplementary Fig. 2F, G, Fig. 4D, E).

Given that verteporfin manifests pleiotropic pharmacological effects by modulating not only the Yap transcription factor, but also by intersecting with diverse molecular pathways, including TEAD-independent signaling and oxidative stress regulators [33, 34], we engineered HC-specific conditional *Yap* knockout (cKO) mice (*Yap* ^*flox/flox*^ *Atoh1* ^*Cre*^) to investigate YAP-specific effects on auditory function (Supplementary Fig. 3A). Western blotting was performed to verify the efficiency of the YAP knockout, and the results showed significantly reduced YAP expression in the basilar membranes of newborn cKO mice (Supplementary Fig. 3B). ABR results showed no significant differences in hearing thresholds between *Yap* ^*flox/flox*^ *Atoh1* ^*Cre*^ mice and *Yap* ^*flox/flox*^ controls at P30, indicating that *Yap* ablation alone did not compromise auditory function in mature mice (Supplementary Fig. 4A–C). To investigate the protective role of YAP against ototoxicity, both cKO and *Yap* ^*flox/flox*^ mice were subjected to neomycin administration (100 mg/kg/day, subcutaneously for 7 days) (Fig. 4F). Post-treatment ABR analysis revealed exacerbated hearing loss in cKO mice, with ABR thresholds significantly higher than those in *Yap* ^*flox/flox*^ controls at frequencies of 4, 8, 12, and 16 kHz (Fig. 4G, H). Quantification of HC loss using myosin 7a staining demonstrated an increase in HC degeneration in cKO mice compared to that in controls, confirming genetic susceptibility to ototoxic injury (Fig. 4I, J). Our experimental results indicate that conditional knockout of YAP exacerbates the outer hair cell damage in the basal and middle turns induced by neomycin, which is consistent with our expectations. We unexpectedly observed that the aggravation of inner hair cell damage was also significant, and this damage was more pronounced in the apical turn. This may be attributed to the higher content of polyunsaturated fatty acids (PUFAs) in this region, which increases the likelihood of metabolic disturbances and the occurrence of ferroptosis [35].

### YAP overexpression can prevent the abnormal LD buildup and HC damage induced by neomycin in vivo

**Fig. 5:**
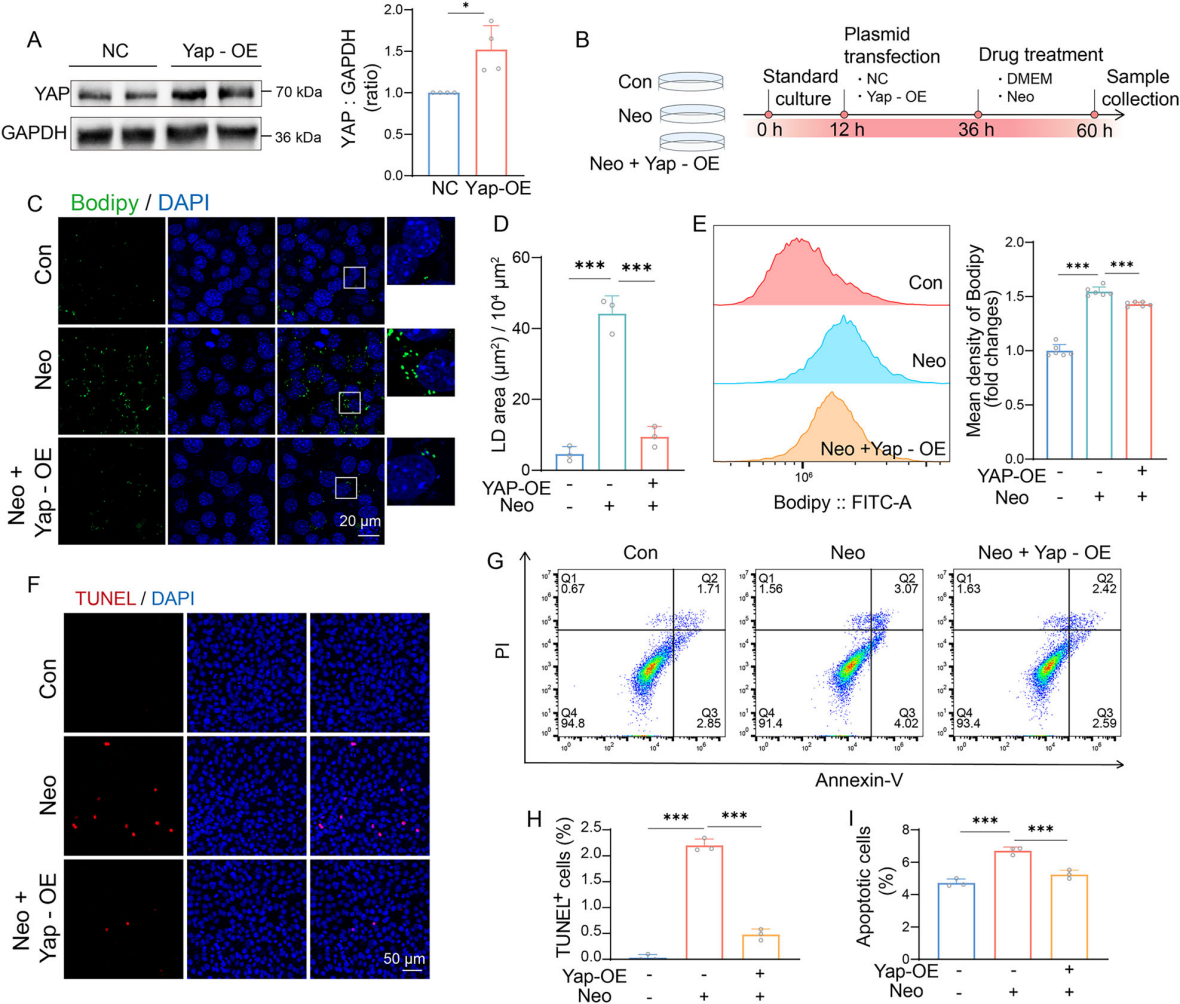
*Yap* overexpression can prevent the abnormal buildup of LD and HC damage induced by neomycin. **A** Western blot analysis and quantification of YAP expression in HEI-OC1 cells after *Yap* overexpression (Yap OE) plasmid transfection. GAPDH was used as an internal reference. *n* = 4. **B** Schematic illustration the experiments of Yap OE plasmid transfection and neomycin administration. **C**,**D** Bodipy (green) and DAPI (blue) staining of HEI-OC1 cells in control, neomycin-only and neomycin + Yap OE group. Scale bars = 20 μm. Data are represented as means ± SD in (**D**). *n* = 3. p value was analyzed by the one-way ANOVA. **E** Flow cytometric analysis of the intensity of bodipy in control, neomycin-only and neomycin + Yap OE groups. *n* = 6. Data was represented as means ± SD. p value was analyzed by one-way ANOVA. **F** Bodipy (red) and DAPI (blue) staining of HEI-OC1 cells in neomycin-only and Yap-OE + neomycin group. Scale bars = 50 μm. **G** The percent of apoptotic cells after treatment with *Yap* overexpression plasmid and neomycin was measured by flow cytometry. **H** Statistical analysis of the proportion of TUNEL+ cells in (**F**). *n* = 3. Data are represented as means ± SD. p value was analyzed by two-way ANOVA. **I** Statistical analysis of the proportion of apoptotic cells in (**G**). *n* = 3. Data are represented as means ± SD. p value were analyzed by the one-way ANOVA. ns = not significant, *p < 0.05, **p < 0.01, and ***p < 0.001.

To investigate whether YAP activation could mitigate neomycin-induced damage in HEI-OC1 cells, we treated the cells with XMU-MP-1, a small-molecule YAP agonist. We evaluated the potential protective effect of XMU-MP-1 against neomycin-induced damage. However, no significant rescue effect was observed (Supplementary Fig. 5A–D). Given the indirect and non-specific effects of XMU-MP-1 on YAP, we directly overexpressed YAP via plasmid transfection. Western blot analysis confirmed successful YAP overexpression in HEI-OC1 cells (Fig. 5A). After HEI-OC1 cells were transfected with *Yap* overexpression plasmid, they were exposed to neomycin for 24 h (Fig. 5B). Bodipy staining results showed that YAP overexpression reduced the neomycin-induced abnormal LD accumulation (Fig. 5C, D). Flow cytometry also showed that *Yap* overexpression reduced the average bodipy fluorescence intensity (Fig. 5E). We then explored the effect of YAP overexpression on HEI-OC1 cell apoptosis after neomycin injury using flow cytometry and TUNEL staining. The results showed that YAP overexpression reduced neomycin-induced apoptosis (Fig. 5F–I). In conclusion, our results indicate that *Yap* overexpression protects against neomycin-induced HC damage by attenuating abnormal LD accumulation.

### YAP overexpression alleviates neomycin-induced LD acccumulation and ototoxicity in vitro

**Fig. 6:**
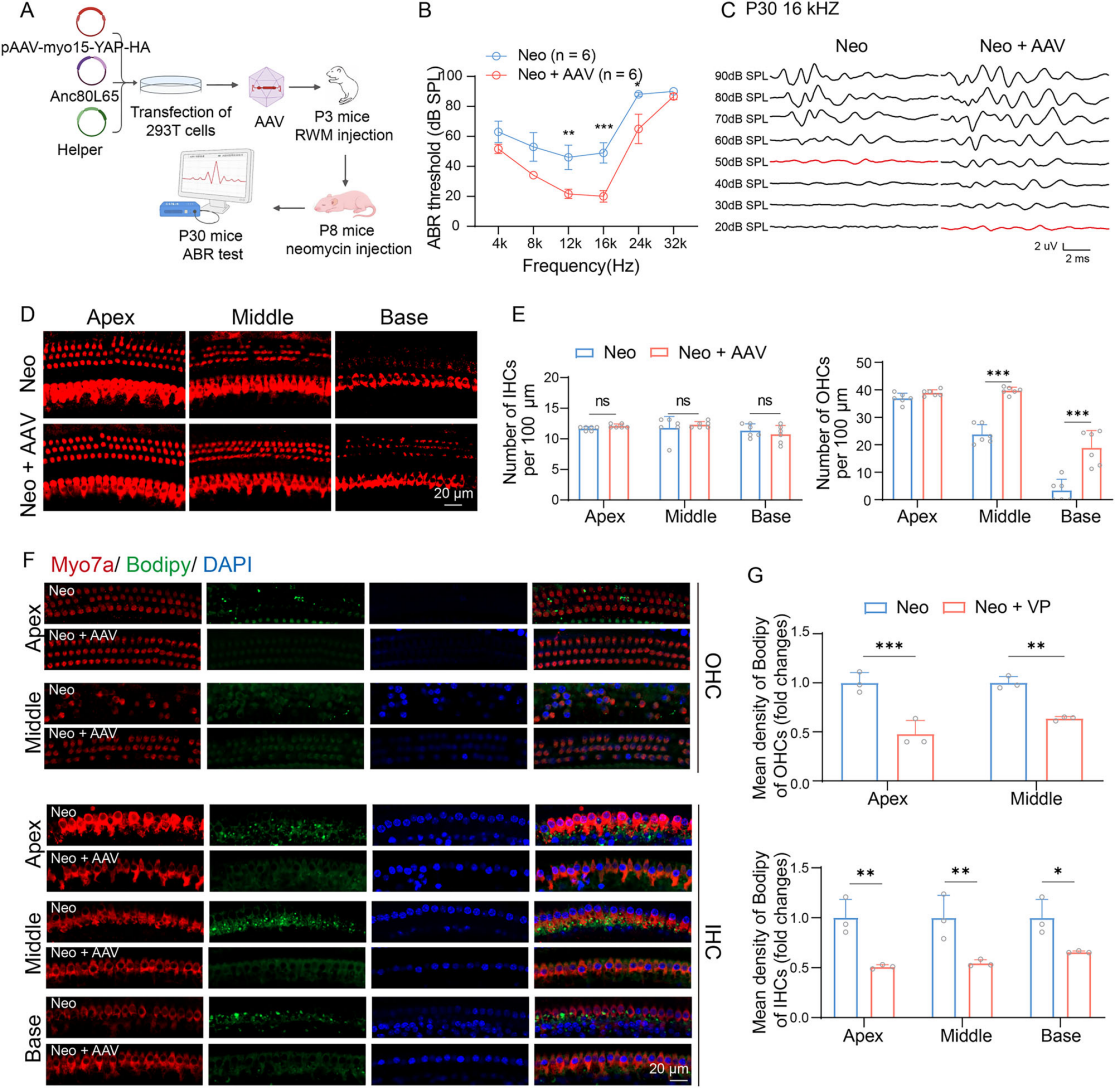
*Yap* overexpression in cochlear HCs alleviates LD accumulation and hearing loss caused by neomycin. **A** Protocol of neomycin and AAV injection into C57 BL/6 mice. **B** ABR thresholds in response to tone-pip stimuli (4, 8, 12, 16, 24, and 32 kHz) in C57 BL/6 mice with treatment of neomycin and AAV injection. Data are represented as means ± SEM. Data from multiple groups was compared by the two-way ANOVA. **C** ABR waveforms at 16 kHz across all sound pressure levels; the red line indicated the ABR thresholds. **D** Immunostaining with myosin7a (red) in apical, middle, and basal turns of cochleae from different groups. Scale bars = 20 μm. **E** Statistical analysis of number of IHCs and OHCs in apical, middle, and basal turns of cochleae in (**D**). *n* = 6. Data are represented as means ± SD. p value was analyzed by the unpaired two-tailed t-test. **F** Immunostaining with myosin7a (red), bodipy (green) and DAPI (blue) in IHCs and OHCs at the apical, middle, and basal turns of cochleae from different groups. Scale bars = 20 μm. **G** Statistical analysis of mean density of bodipy in IHCs and OHCs. *n* = 3. Data are represented as means ± SD. p value was analyzed by the two-way ANOVA. ns = not significant, *p < 0.05, **p < 0.01, and ***p < 0.001.

To investigate the protective potential of YAP overexpression against aminoglycoside-induced ototoxicity, we constructed a YAP-overexpression adeno-associated virus (AAV). The AAV was packaged using Anc80L65 capsid, with *Yap* expression driven by HC-specific promoter Myo15 and fused with an hemagglutinin (HA) tag. P3 C57BL/6 J mice received unilateral intracochlear AAV injection via the round window membrane, while control mice received PBS injections. From P8 to P14, the mice received daily subcutaneous injections of 150 mg/kg neomycin, followed by ABR measurements and immunostaining at P30 (Fig. 6A). According to literature reports, when AAVs are injected through the round window membrane in mice at P2–P4, serotype Anc80 successfully transduces both inner and outer hair cells within 5 days post-injection [36, 37]. Confocal imaging revealed robust HA expression selectively in the outer and inner HC after AAV injection, with no detectable HA signal in the PBS-injected cochleae of P30 mice (Supplementary Fig. 6A). These results indicated that AAV can effectively and specifically infect HC. Compared to controls, AAV-injected cochleae exhibited significantly attenuated hearing loss following neomycin injection (Fig. 6B, C). The immustaining results demonstrated more HC in AAV-injected cochlea than in PBS-injected cochlea (Fig. 6D, E). This proof-of-concept study, compliant with Nature Gene Therapy standards (doi:10.1038/s41434-023-00411-3), establishes *Yap* gene augmentation as a viable strategy to mitigate aminoglycoside ototoxicity while preserving baseline hearing, which is a critical advance in targeted inner ear gene therapies.

LD in cochlear HC were detected using myosin7a and bodipy co-staining. The results showed that AAV treatment reduced the neomycin-induced accumulation of LD in the IHC and OHC of the apical, middle, and basal turns of the cochlea (Fig. 6G). Notably, basal-turn OHC were excluded from the analysis owing to near-complete loss of neomycin-induced ototoxicity.

### YAP alleviates neomycin-induced LD accumulation by upregulating COX2

**Fig. 7:**
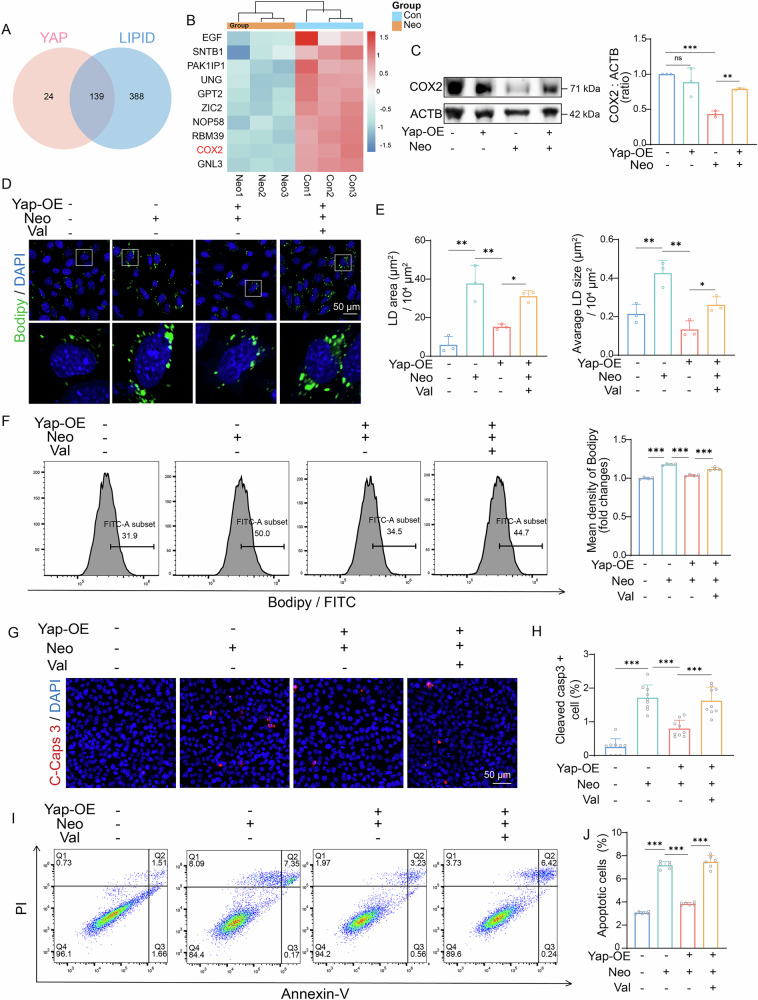
Yap alleviates the accumulation of LDs caused by neomycin by upregulating COX2. **A** Venn diagram analysis of Yap-related and lipid-related gene in differentially expressed genes. **B** Cluster heatmap of the top ten cross-genetic factors. **C** Western blot analysis the expression of COX2 in HEI-OC1 cells of different group. ACTB was used as an internal reference. *n* = 3. Data are shown as means ± SD. p value was analysed by the one-way ANOVA. **D**,**E** Bodipy (green) and DAPI (blue) staining of HEI-OC1 cells in control, neomycin-only, valdecoxib-only and neomycin + valdecoxib group. treated groups. Scale bars = 50 μm. *n* = 3. Data are represented as means ± SD in (**E**). p value was analyzed by the one-way ANOVA. **F** Flow cytometry was used to detect the proportion of apoptotic cells. *n* = 4. Data are shown as means ± SD. p value was analyzed by the one-way ANOVA. **G**,**H** Cleaved-caspase 3 (red) and DAPI (blue) double staining shows the proportion of apoptotic HEI-OC1 cells after different treatments. Scale bars = 50 μm, *n* = 9. Data are represented as means ± SD. p value was analyzed by the one-way ANOVA. **I**,**J** Flow cytometry was used to detect bodipy immunofluorescence intensity. *n* = 6. Data are shown as means ± SD. p value was analyzed by the one-way ANOVA. ns, not significant, *p < 0.05, **p < 0.01, and ***p < 0.001.

While our previous studies established the role of YAP in suppressing LD accumulation during neomycin treatment, the precise downstream effectors mediating this metabolic regulation remain undefined. To address this, we analyzed the RNA sequence data from neomycin-treated HEI-OC1 cells. Venn analysis identified 139 candidate genes at the intersection of *Yap* transcriptional targets and lipid regulatory pathways (Fig. 7A). We have listed the top ten genes that are downregulated after neomycin treatment, among which *Cox2* emerged as a prioritized node, given its dual annotation as a reported *Yap* transcriptional target and key regulator of arachidonic acid metabolism (Fig. 7B). Western blotting was performed to validate the regulation of COX2 expression by YAP and neomycin. The results showed that neomycin exposure significantly suppressed COX2 expression, whereas YAP overexpression effectively rescued COX2 levels (Fig. 7C).

**Fig. 8:**
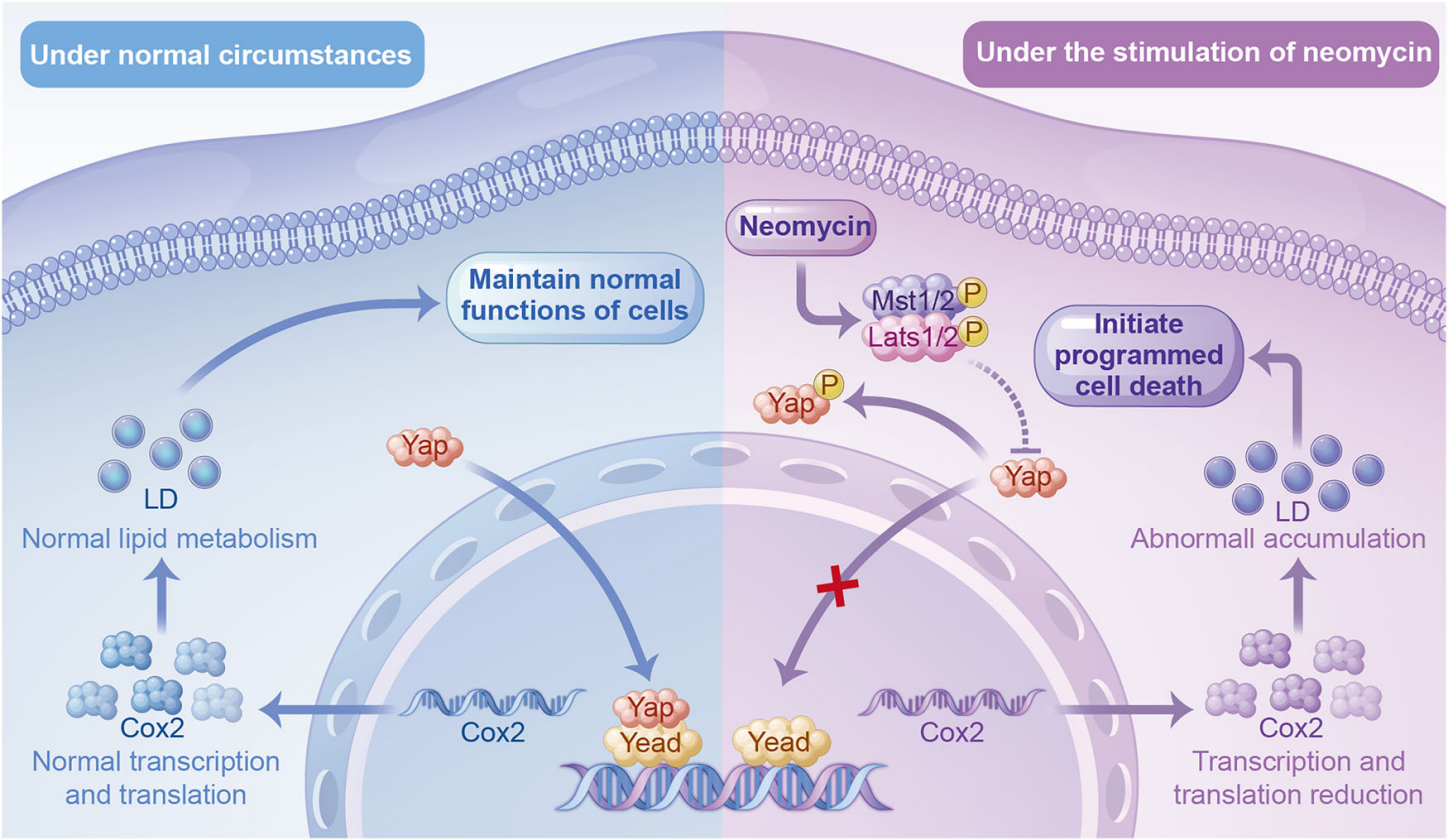
Schematic diagram of the Hippo-Yap/COX2 signaling pathway. Under normal conditions, Yap enters the cell nucleus and binds to TEAD to promote the transcription and translation of COX2. COX2 plays a crucial role in lipid metabolism and helps maintain normal cell functions. When stimulated by neomycin, the Hippo signaling pathway is activated, leading to the phosphorylation and subsequent degradation of Yap in the cytoplasm. As a result, the transcription and translation of COX2 are reduced, leading to lipid metabolism disorders, abnormal accumulation of lipid droplets, and ultimately, cell death.

Valdecoxib is a COX2 inhibitor. Its mechanism of action involves rapidly forming additional hydrogen bonds with the hydrophobic pocket adjacent to the COX-2 active site, resulting in extremely slow dissociation and near-irreversible inactivation [38]. After culturing the cells for 12 h, we transfected the YAP overexpression plasmid. After 24 hours, neomycin and valdecoxib were added. Samples were collected 24 h after the drug treatment. Immunofluorescence staining and flow cytometry showed that the inhibition of COX2 by valdecoxib abolished the beneficial effect of YAP overexpression on the neomycin-induced abnormal LD accumulation (Fig. 7D–F). The number of apoptotic cells in the neomycin + YAP overexpression + valdecoxib group was higher than that in the neomycin + YAP overexpression group (Fig. 7G–J). These findings collectively delineate COX2 as a critical downstream effector of YAP-mediated metabolic regulation. Specifically, YAP sustains cochlear lipid homeostasis under ototoxic stress by maintaining COX2 expression (Fig. 8). This discovery not only resolves a key gap in understanding the downstream network of YAP, but also positions COX2 modulators as potential adjuvants to enhance YAP-targeted therapies against aminoglycoside-induced hearing loss.

## Discussion

Lipids serve multiple essential functions within cells, encompassing energy storage, preservation of membrane integrity, signal transduction, and hormone biosynthesis, among other roles[39]. LD, as important lipid storage sites, are composed of TGs and cholesterol at the core surrounded by a phospholipid monolayer and LD-related proteins (mainly members of the perilipin family) [40]. LD exist in most organisms and cell types, not only as lipid storage containers but also as participants in various complex cellular functions. The lipids stored in LD are broken down into free fatty acids (FAA), which not only provide energy for the cells but also regulate other cellular processes, activate cell signaling pathways, or cause lipid toxicity [41]. The abnormal LD accumulation caused by neomycin leads to lipid toxicity, which ultimately results in HC damage.

We observed LD accumulated in neomycin-treated HEI-OC1 cells. In the 1980s, LD were first reported in Hensen cells. Intense noise exposure triggers the release of LD from Hensen cells in guinea pigs. The membrane-linked protein ANXA1, found in LD, is released into the extracellular environment and regulates cell death signaling and the clearance of apoptotic cells by phagocytes[42]. Research on LD in various hearing-loss models and auditory cells has significantly expanded in recent years. In an age-related hearing loss (ARHL) model, nicotinamide riboside (NR), a precursor of NAD^+^, alleviated ARHL onset by influencing proteins related to the growth of LD[43]. In aged APOE4 knockout mice, abnormal LD accumulation was observed in spiral ganglion neurons (SGN) accompanied by damaged lipophagy and axonal demyelination[44]. These findings confirm the significant role of LD in the onset and progression of hearing loss.

Whether lipid droplet accumulation serves a protective role for cells depends on the extent and duration of accumulation, as well as the intracellular metabolic environment. In our experimental results, lipid droplet levels initially increased, then declined. A possible explanation is that lipid droplets play different roles depending on the severity of cellular damage. Under mild damage, cellular metabolism is disrupted, leading cells to esterify excess, harmful lipids such as fatty acids and cholesterol, and sequester them into lipid droplets. This prevents these harmful substances from damaging other organelles. Meanwhile, these lipid droplets can be metabolized by neutral or acidic lipases in lysosomes to provide energy to mitochondria or to be converted into lipid signaling molecules [45]. In this scenario, lipid droplet accumulation remains within a short-term and controllable range, representing a protective response. However, excessive lipid droplet accumulation can disrupt intracellular lipid metabolism homeostasis, exacerbate endoplasmic reticulum stress and mitochondrial dysfunction, and even trigger programmed cell death [35].

YAP is involved in HC damage caused by various drug toxicities during the occurrence and development of SNHL [16, 17]. In this study, we explored the potential of YAP as a therapeutic target for aminoglycoside-induced SNHL, and confirmed that YAP participates in cell death by regulating lipid metabolism. Studies have shown that the YAP agonist LAT1-IN-1 can mitigate cisplatin-induced damage in HEI-OC1 cells by reducing the activation of ferroptosis [17]. Significant changes in the Hippo signaling pathway have also been observed in enrichment analyses of age-related hearing loss (ARHL) databases [46]. In a Mendelian randomization analysis, LATS1 was identified as a drug target significantly associated with sensorineural hearing loss (SNHL). As a key protein in the Hippo signaling pathway, LATS1 primarily acts on its downstream target YAP [47]. Lipids also play important roles in programmed cell death. Palmitate can trigger endoplasmic reticulum stress and initiating apoptosis signals [48]. Sphingolipids (SP) act as mediators of apoptosis by oligomerizing on the outer mitochondrial membrane to form the BAK/BAX protein [49]. Necroptosis requires a combination of membrane phosphatidylinositol phosphate (PIP) and mixed lineage kinase domain-like protein (MLKL), leading to membrane leakage and ion homeostasis imbalance [50]. Fatty acid synthesis and mevalonate flux regulate the sensitivity of cells to ferroptosis via different mechanisms [51]. Further, there is evidence suggesting a link between aminoglycoside toxicity and the abnormal LD accumulation and lipid toxicity [52–54].

The primary substrates for cyclooxygenases (COX-1 and COX-2) include arachidonic acid (AA), phospholipids released through the activity of phospholipase A2 (PLA2), and endogenous cannabinoids generated via the enzymatic actions of fatty acid amide hydrolase (FAAH) and monoacylglycerol lipase (MAGL) [55]. In colorectal cancer cells, YAP and COX2 show positive correlations. It has been reported that YAP, as an upstream regulatory factor of COX2, plays a role in regulating COX2 transcriptional levels [25]. This finding was confirmed in the present study. In this study, the expression of both YAP and COX2 under neomycin-induced injury showed a decreasing trend. After supplementation with YAP, COX2 protein levels showed a corresponding increase. However, the application of COX2 inhibitors hindered the salvage effect of YAP on neomycin-induced injury.

In this study, we demonstrated the role of lipid metabolism in the auditory damage caused by neomycin. Through neomycin-induced injury models in vitro and in vivo, we proved that YAP has a protective effect on hair cells. Verteporfin exacerbated the HC damage caused by neomycin. Although XMU-MP-1 treatment failed to protect HC from neomycin-induced damage, we rescued the neomycin-induced HC damage and hearing loss by overexpressing YAP. This might be because of the limited efficacy of small-molecule agonists, which are compensated for by other intracellular signaling pathways. Our findings also demonstrated that YAP modulated the expression of the downstream target COX2, thereby affecting lipid accumulation during neomycin-induced injury. These findings may offer novel therapeutic targets for the prevention and treatment of SNHL caused by aminoglycoside antibiotics.

## Materials and methods

### Mice treatment

Experiments were conducted using both male and female C57BL/6 J mice, which were procured from Jangsu Qinglongshan Biotechnology Co., Ltd. (Jangsu, China). All experimental procedures received approval from the Animal Care and Use Committee of Nanjing University and were performed in accordance with the National Institutes of Health Guide for the Care and Use of Laboratory Animals. For in vivo investigations, mice were administered daily subcutaneous injections of neomycin (at doses of 100 mg/kg or 150 mg/kg; Sigma-Aldrich, Shanghai, China, N6386), verteporfin (1 mg/kg; Sigma-Aldrich, ML0534), or sterile saline over a period of seven days, as specified.

Yap-flox and Atoh1-Cre transgenic mice were obtained from Cyagen Biosciences. Conditional knockout (cKO) of Yap in HC was achieved by crossing Yap-flox mice with Atoh1-Cre transgenic mice, wherein Cre recombinase expression is driven by the Atoh1 promoter to facilitate HC-specific recombination. Genotyping of transgenic mice was performed using genomic DNA extracted from tail biopsies. DNA extraction involved incubation of tail tips in 90 µL of 50 mM NaOH at 98 °C for 1 h, followed by neutralization with 10 µL of 1 M Tris-HCl (pH 7.0). The primers employed for genotyping were as follows: Yap-flox: (F) 5’- GGG AAA CTG GTA CTT ACT AGG TGA A-3’; (R) 5’- CAC CAG CCT TTA AAT TGA GAA C-3’. Atoh1-Cre: (F) 5’-GCT TAA TCT TCA CAA AGG GGT AGT-3’; (R) 5’-GCA AAC GGA CAG AAG CAT TTT CC-3’. Polymerase chain reaction was conducted using 3 µL of genomic DNA, 2 µL of primer mix, 10 µL of 2× PCR mix (Vazyme, Nanjing, Jiangsu, China, P131), and nuclease-free water to a final volume of 20 µL. The thermal cycling protocol comprised an initial denaturation at 95 °C for 3 min, followed by 38 cycles of denaturation at 95 °C for 30 s, annealing at 60 °C for 30 s, and extension at 72 °C for 30 s.

### Cell culture

HEI-OC1 cells were procured from the American Type Culture Collection. These cells were maintained in Dulbecco’s Modified Eagle’s Medium (Vivacell, Shanghai, China, C3110) supplemented with 10% (v/v) fetal bovine serum (Vazyme, F101) and 1% (v/v) ampicillin (100 mg/mL; Sangon Biotech, Shanghai, China, A100339-0005). All cell cultures were incubated at 37 °C in an atmosphere containing 5% CO_2_.

### Cell viability assay

Cell viability was assessed using the Cell Counting Kit-8 (CCK-8; Beyotime, Shanghai, China, C0038) in accordance with the manufacturer’s protocol. In brief, cells were plated in triplicate at a density of 5000 cells per well in 96-well plates and incubated overnight under standard culture conditions. Following drug administration, 100 µL of fresh culture medium supplemented with 10% CCK-8 reagent was added to each well, and the plates were incubated for an additional 1 hour. Subsequently, the optical density (OD) was measured at 450 nm using a microplate reader (Bio-Rad, Hercules, CA, USA). A blank control group, which underwent the same procedure without cell seeding, was included, alongside a control group that did not receive any drug treatment. Relative cell viability was calculated using the following formula:

$${\rm{Relativeviability}}=({{\rm{A}}}_{{\rm{experiment}}}{-{\rm{A}}}_{{\rm{blank}}})/({{\rm{A}}}_{{\rm{control}}}{-{\rm{A}}}_{{\rm{blank}}})\times 100$$

where A represents absorbance.

### Flow cytometry analysis

HEI-OC1 cells were seeded at a density of 5 × 10^5^ cells per well in six-well plates and incubated overnight. Following trypsinization, the cells were harvested for analysis. Apoptotic cell populations were quantified using the Annexin V/PI Apoptosis Detection Kit (Yeasen, Shanghai, China, 40302ES20). Fluorescence signals were acquired on a BD FACSAria II flow cytometer (BD Biosciences, San Jose, CA, USA). For each sample, 10,000 events were recorded to ensure adequate statistical representation. Data processing and analysis were conducted using FlowJo software, with polygonal gating strategies employed to exclude cellular debris from the analysis.

### Cochlear explant culture

Cochleae were rapidly dissected from postnatal day 3 (P3) C57BL/6 mice in ice-cold phosphate-buffered saline (PBS; Solarbio, Beijing, China, D1040) and subsequently maintained in four-well culture plates containing DMEM (Vivacell, C3110). The culture medium was supplemented with 1% N2 (Stemcell, Vancouver, BC, Canada, 07152), 2% B27 supplement (Stemcell, 05711), and 50 IU/mL penicillin (Gibco, Waltham, MA, USA, 15140122). Following an initial 24-hour culture period, cochlear explants were pre-incubated for an additional 24 h at 37 °C in a 5% CO_2_ atmosphere, using culture medium either with or without neomycin. Subsequently, cochlear explants were co-incubated with 10 mmol/L neomycin. Verteporfin was administered at a concentration of 0.4 µmol/L, a dosage determined based on prior dose-response assessments.

### Virus packaging and purification

A plasmid was engineered to include the Myo15 promoter, the coding sequence of Yap, a polyadenylation signal, and additional enhancer elements. An HA epitope tag was inserted at the 3’ terminus of the Yap coding region. Adeno-associated virus (AAV) vectors were generated using the Anc80L65 serotype. Viral packaging was performed in HEK293T cells cultured in medium supplemented with 1% (v/v) penicillin/streptomycin (Gibco, 15140122). Forty-eight hours post-transfection with the plasmids, supernatants were collected, and both the culture media and cells were harvested at 96 hours. Viral particles in the medium were concentrated using polyethylene glycol 8000 (PEG 8000; Aladdin, Shanghai, China, p274350) and sodium chloride (NaCl; Sigma-Aldrich, S9888). The resulting precipitate was resuspended in a solution containing DNase I (10 U/mL; Thermo Fisher, Waltham, MA, USA EN0525) and RNase A (10 U/mL; Thermo Fisher, EN0531) supplemented with magnesium chloride (MgCl2; Thermo Fisher, B43) and incubated at 37 °C for one hour. Subsequent purification of AAV was achieved through iodixanol gradient ultracentrifugation with layers of 15, 25, 40, and 60% iodixanol. The genomic titer of the AAV preparations was quantified by fluorescence-based quantitative PCR, employing linearized plasmid DNA as a standard and primers targeting the inverted terminal repeat sequences.

For in vivo delivery, AAV was administered to neonatal mice via round-window membrane injection. Neonatal mice were anesthetized by immersion in ice, followed by a small incision at the junction of the ear and neck using ophthalmic scissors. Sterile forceps were used to gently retract adipose and muscle tissues to expose the round window membrane. Viral particles (1 µL at a concentration of 1 × 10^13^ vector genomes per milliliter) were injected using a glass micropipette. After injection, the displaced tissues were repositioned, and the incision site was sealed with a tissue adhesive (Vetbond, St. Paul, MN, USA, 1469SB) to prevent postoperative disruption. Mice were then placed on a heating pad maintained at 37 °C until full recovery, after which they were returned to their home cages.

### Immunostaining and confocal microscopy

For neonatal mice aged P0-P7, cochleae were carefully dissected using fine forceps (World Precision Instruments) in cold Hank’s Balanced Salt Solution (HBSS) and subsequently fixed in 4% paraformaldehyde (Biosharp, Beijing, China, BL53A) for one hour at room temperature (RT). In contrast, cochleae from mice older than P7 were initially fixed in 4% paraformaldehyde (Beyotime, P0099) for one hour at RT, followed by decalcification in 0.5 M ethylenediaminetetraacetic acid (Beyotime, C196) for a duration of 1–3 days, depending on the animal’s age, also at RT. After decalcification, these cochleae were dissected in HBSS.

Subsequent to fixation and dissection, all samples were rinsed with PBS and subjected to a blocking step. The blocking solution comprised 5% donkey serum, 0.5% Triton X-100, 0.02% sodium azide, and 1% bovine serum albumin, adjusted to pH 7.4. Blocking was conducted for one hour at RT. Primary antibodies were diluted in PBS containing 2.5% donkey serum, 0.1% Triton X-100, 0.02% sodium azide, and 1% BSA, then applied to the samples, which were incubated overnight at 4 °C. Following primary antibody incubation, samples were washed three times with PBS containing 0.1% Triton X-100 (pH 7.4). Fluorescently conjugated secondary antibodies (Invitrogen, Carlsbad, CA, USA) were diluted 1:400 in PBS with 0.1% Triton X-100 and 1% BSA, applied to the samples, and incubated for one hour at RT. Finally, samples were mounted on glass slides using DAKO mounting medium (Protein Biotechnologies, Ramona, CA, USA, S3023), and imaging was performed with a Zeiss LSM900 confocal microscope. The primary antibodies employed included anti-Myosin7a (Proteus Bioscience, 25-670), anti-YAP (Cell Signaling Technology, Danvers, MA, USA, 14074 T), and anti-HA (Cell Signaling Technology, 3724S).

### Bodipy staining

Cells or tissue samples were incubated with 2 μg/mL bodipy 493/503 (Amgicam, Wuhan, China, ajci70160) at 37 °C for 30 min in the dark. Subsequently, the cultured cells were washed 3 times with precooled PBS, each wash lasting 5 min, followed by fixation with 4% paraformaldehyde for 30 min. Nuclear staining was then performed using DAPI (Solarbio, C0060) at 37 °C for 20 min. Lipid droplets were visualized using fluorescence confocal microscopy.

### Western blot

Whole-cell lysates of HEI-OC1 cells and cochlear tissues were prepared using RIPA lysis buffer (Beyotime, P0013B) supplemented with a protease inhibitor cocktail (Sigma, 11674998001). The lysates were subjected to centrifugation at 12,000 g for 10 min at 4 °C, and the resulting supernatants were collected for subsequent protein analysis. Protein concentrations were quantified using the BCA Protein Assay Kit (Beyotime, P0010S). Equal amounts of protein from each lysate were resolved by sodium dodecyl sulfate-polyacrylamide gel electrophoresis and transferred onto polyvinylidene difluoride membranes (Millipore, Burlington, MA, USA IPVH00010). Immunoblotting was performed using the following primary antibodies: anti-cleaved caspase-3 (Cell Signaling Technology, 9664S), anti-YAP1 (ABclonal, Wuhan, China, A1001), and anti-COX2 (Abclonal, A7515). Membranes were subsequently washed three times with TBST for 10 min each and incubated with horseradish peroxidase-conjugated secondary antibodies (diluted 1:5000) at 37 °C for 1 h. Protein bands were visualized using the SuperSignal West Dura chemiluminescent substrate kit (Thermo Fisher, 34580) and scanned for analysis. Band intensities were quantified relative to the loading controls ACTB or GAPDH using ImageJ software. Full length western blots are provided in the supplementary materials.

### RNA isolation and qPCR

Cochlear explants or HEI-OC1 cells were combined for RNA isolation, with total RNA extracted utilizing the TRIzol reagent (Invitrogen, 15596026). The concentration and purity of the extracted RNA were assessed via a NanoDrop 2000 spectrophotometer (Thermo Fisher). The absorbance ratios at 260/280 nm for all samples ranged from 1.8 to 2.0, indicating acceptable RNA quality. Subsequently, total RNA was reverse-transcribed into complementary DNA employing Superscript III reverse transcriptase (Vazyme, R333). Quantitative polymerase chain reaction analyses were conducted using an ABI 7500 Real-Time PCR System (Applied Biosystems, Foster City, CA, USA). The primer sequences utilized in this investigation are detailed in Supporting Information Table S1. Actin served as the endogenous reference gene. Each qPCR assay was performed in triplicate, and relative gene expression levels were calculated using the 2^−ΔΔCt^ method.

### ABR measurements

ABR measurements were conducted utilizing a Tucker-Davis Technologies System III (TDT, Gainesville, FL, USA). All mice were anesthetized during the assessments. To maintain physiological body temperature throughout the procedure, a heating pad was employed. Electrical brain activity elicited by auditory stimuli was recorded via electrodes, capturing closed-field ABR responses. The auditory stimuli encompassed six frequencies spanning from low to high: 4, 8, 12, 16, 24, and 32 kHz. Recorded signals were amplified by a factor of 10,000, band-pass filtered between 0.1 and 3 kHz, averaged, and subsequently processed using BioSigRZ software (TDT). Sound pressure levels (SPL) were systematically reduced from 90 dB to 0 dB in 5 dB increments. At each SPL, an average of 1024 responses with alternating stimulus polarity were collected. The auditory threshold was operationally defined as the lowest SPL at which a discernible ABR waveform was detected.

### Statistical analysis

A minimum of three independent replicates were conducted for each experimental condition. Data analysis was performed using GraphPad Prism 8 software, with results expressed as means ± standard errors. For comparisons between two groups, statistical significance was assessed using two-tailed unpaired Student’s t-tests. When comparing more than two groups, one-way or two-way analysis of variance (ANOVA) was employed, followed by Bonferroni post-hoc tests. A p-value of less than 0.05 was considered indicative of statistical significance.

## Supplementary information

Supplementary figure

original western blots

## Data Availability

The materials described in the manuscript, including all relevant raw data, will be freely available to any researcher wishing to use them for non-commercial purposes, without breaching participant confidentiality.
