# Supplementary figures and images for: Regulation of lipid droplets accumulation by the Hippo–YAP/COX2 signaling pathway in neomycin-induced ototoxicity
Source: Cell Death Discov. 2026 Apr 16;12:248. doi: 10.1038/s41420-026-03115-w (PMC13201534; doi:10.1038/s41420-026-03115-w)

Figure 2F


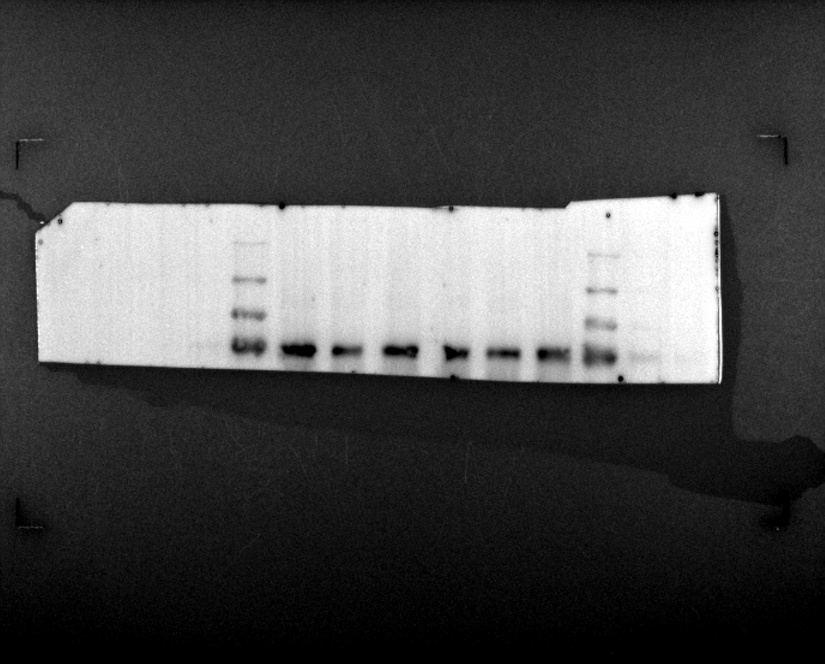


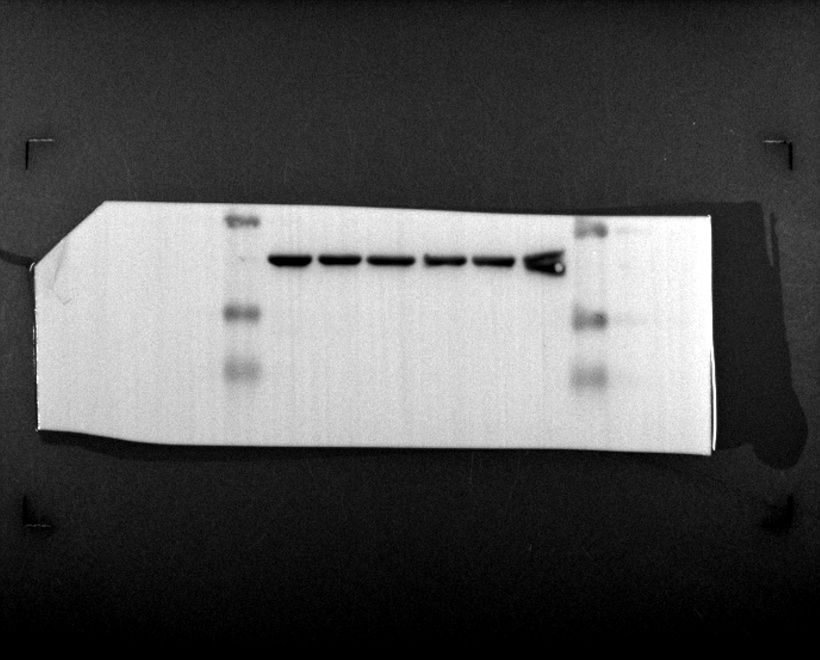


Figure 5A


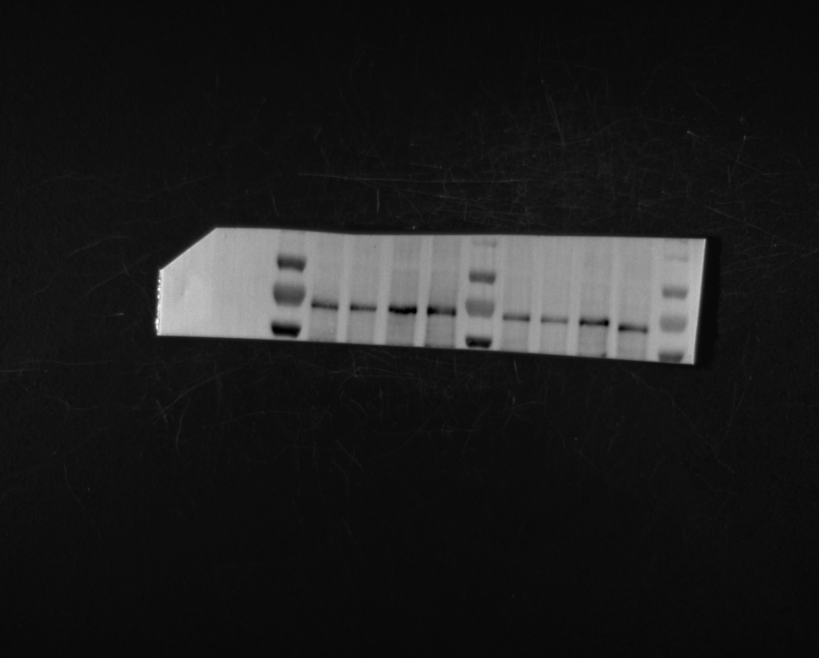


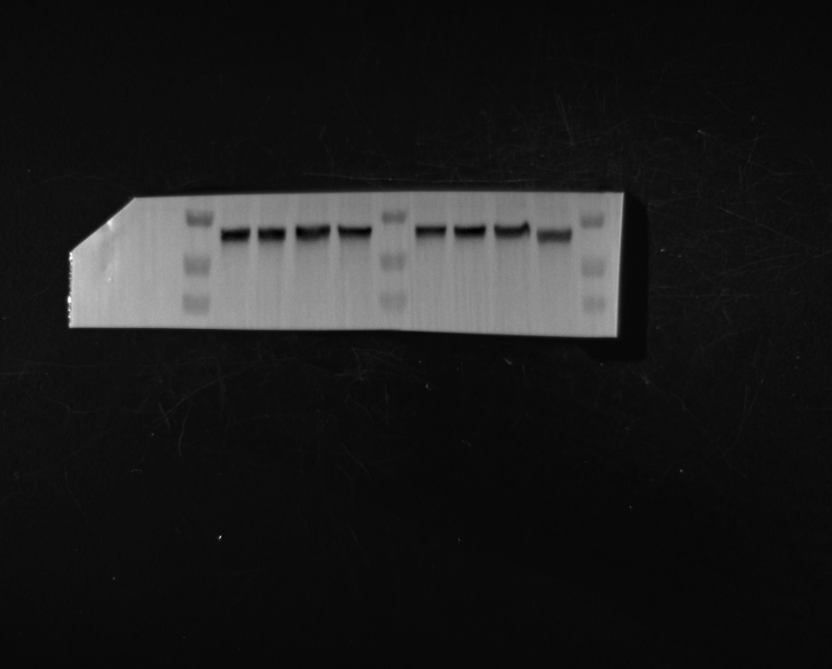


Figure 7C


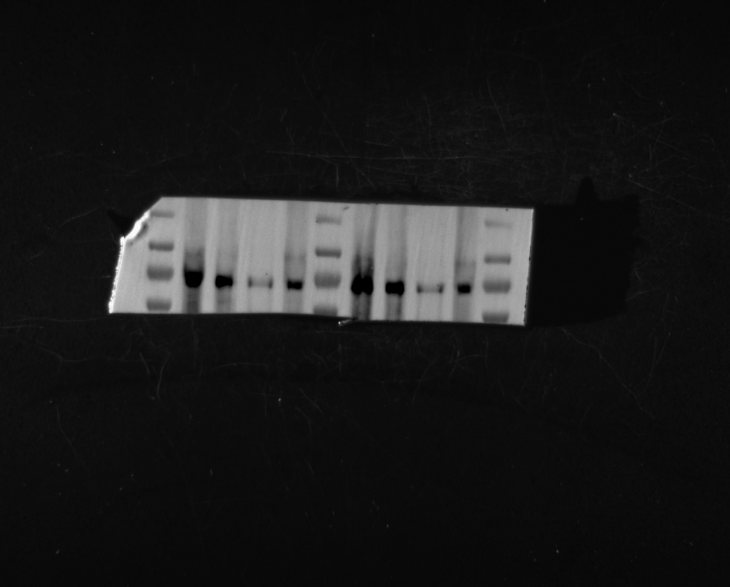


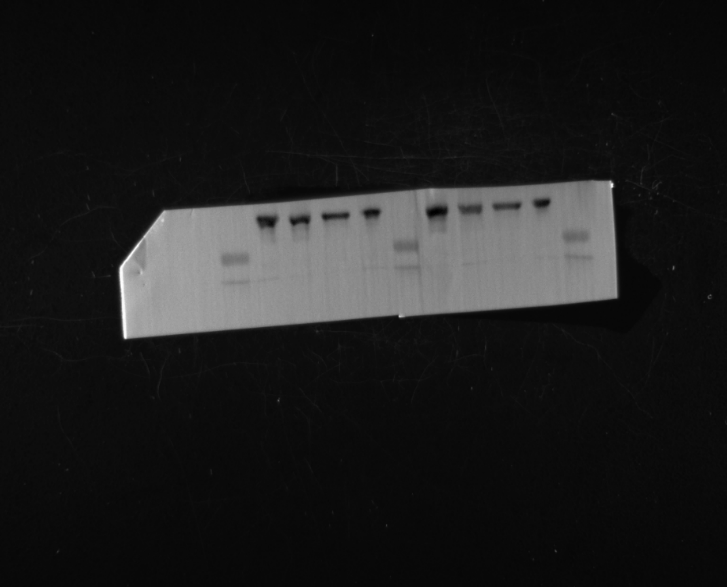


SF 1D


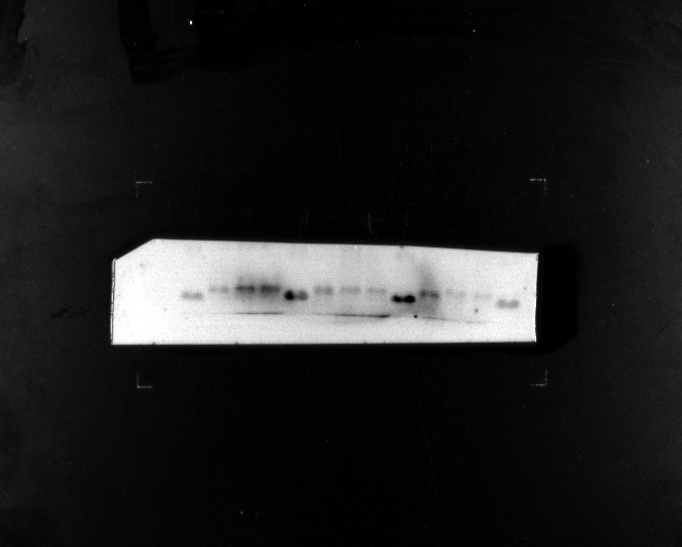


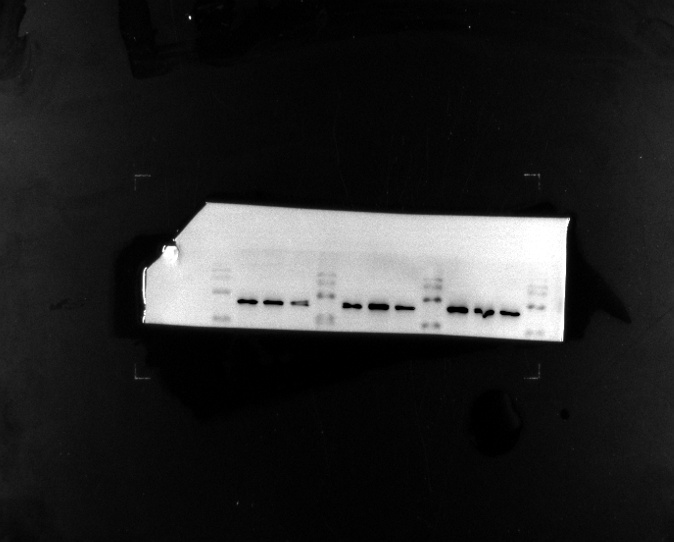


SF 4B


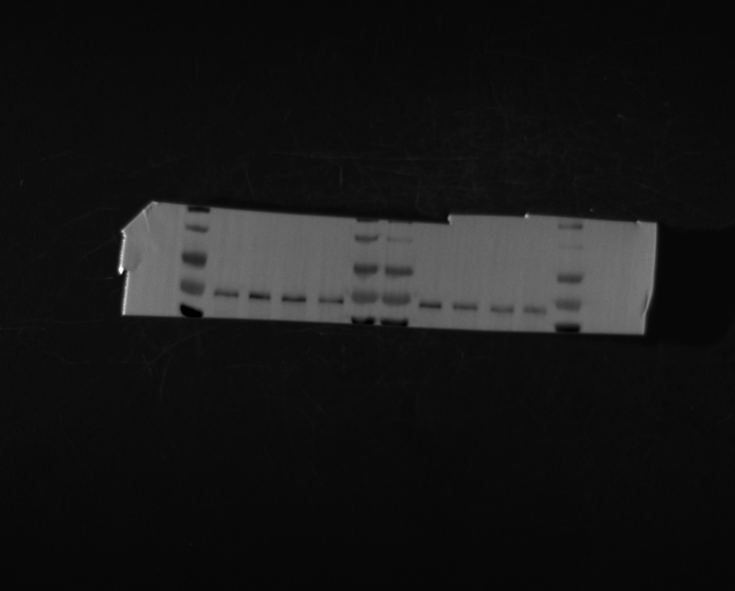


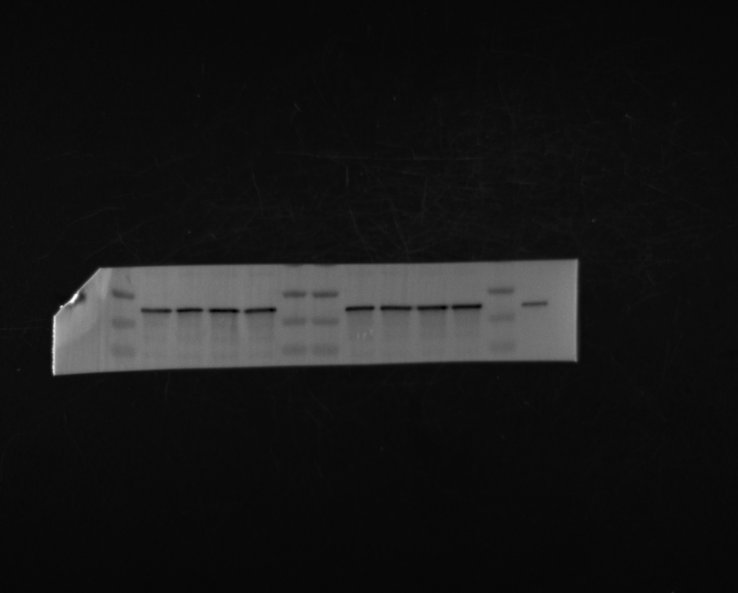

Supplement: Supplementary file 2 — original western blots [file 41420_2026_3115_MOESM2_ESM.docx]
