# Supplementary material for: Regulation of lipid droplets accumulation by the Hippo–YAP/COX2 signaling pathway in neomycin-induced ototoxicity
Source: Cell Death Discov. 2026 Apr 16;12:248. doi: 10.1038/s41420-026-03115-w (PMC13201534; doi:10.1038/s41420-026-03115-w)
Supplement: Supplementary file 1 — Supplementary figure [file 41420_2026_3115_MOESM1_ESM.docx]

Title: Regulation of lipid droplets accumulation by the Hippo–YAP/COX2 signaling pathway in neomycin-induced ototoxicity

Wenli Hao^1^, Suhan Guo^1^, Song Gao^1^, Siyu Li^1^, Junze Lu^1^, Lulu Jiang^2^, Jie Lu^3^, Jingjing Luo^1^, Nan Wang^1^, Aijia Ran^1^, Xiaoyun Qian^1, 4^*, Xia Gao^1, 4^*, Chenjie Yu^1, 4^*, Cheng Cheng^1, 4, 5^*

**Supplementary figure 1.** The inhibition of Yap aggravated HEI-OC1 damage caused by neomycin. (A) ABR thresholds in response to tone-pip stimuli (4, 8, 12, 16, 24, and 32 kHz) in C57 BL/6 mice with treatment of neomycin. (B) HEI-OC1 cells were treated with verteporfin for 24 h. Cell viability was then determined CCK8 assays. n = 5. Data are shown as means ± SD. Data were analyzed by the one-way ANOVA. (C) The qPCR assay was used to investigate the effect of verteporfin on the transcriptional levels of the downstream target genes *Ankrrd1*, *Birc5* and *Ccn2* in response to neomycin treatment. Data are represented as means ± SD and analyzed by the unpaired two-tailed t-test. (D) Western blot analysis the expression of Cleaved-caspase 3 in HEI-OC1 cells after neomycin and verteporfin treatment. ACTB was used as an internal reference. (E) Cleaved-caspase 3 (red) and DAPI (blue) double staining showing apoptotic HEI-OC1 cells after neomycin-only and neomycin + VP treatments. Scale bar = 50 μm. (F) Statistical analysis the proportion of caspase 3+ cells in (E). Data are shown as means ± SD. n = 6. Data were analyzed by the one-way ANOVA. ns = not significant, *p < 0.05, **p < 0.01, and ***p < 0.001.

**Supplementary figure 2.** Verteporfin exacerbated neomycin-induced hearing loss and HC loss by inhibiting YAP function. (A) ABR thresholds in response to tone-pip stimuli (4, 8, 12, 16, 24, and 32 kHz) in C57 BL/6 mice with treatment of verteporfin injection or PBS injection. (B) Immunostaining with myosin7a (red) in apical, middle, and basal turns of cochleae from control, neomycin-only, and neomycin + verteporfin. Scale bars = 20 μm. (C) Statistical analysis of HC Counting in (B). n = 6. Data are represented as means ± SD. Data were analyzed by the two-way ANOVA. (D) The transcriptional levels of *Yap* downstream target genes *Ankrrd1*, *Birc5* and *Ccn2* in C57 BL/6 mice after treated with verteporfin. Data are represented as means ± SD and analyzed by the unpaired two-tailed t-test. (E) ABR thresholds in response to tone-pip stimuli (4, 8, 12, 16, 24, and 32 kHz) in P15 C57 BL/6 mice with treatment of verteporfin and neomycin injection. (F) Immunostaining with myosin7a (red) in apical, middle, and basal turns of cochleae from different groups. Scale bars = 20 μm. (G) Statistical analysis of OHC and IHC in (F). n = 6. Data are represented as means ± SD and analyzed by the unpaired two-tailed t-test. ns = not significant, *p < 0.05, **p < 0.01, and ***p < 0.001.

**Supplementary figure 3.** Verification of effective Yap knockoutin cKO mice cochlear. (A) Breeding scheme to generate conditional KO mice and genotyping strategy for conditional KO mice. (B) Western blot analysis the expression of YAP in cKO and wild-type mice cochlea. GAPDH was used as an internal reference. n = 3. *p < 0.05.

**Supplementary figure 4.** The knockout of Yap does not affect the hearing of mice. (A) ABR thresholds in response to tone-pip stimuli (4, 8, 12, 16, 24, and 32 kHz) in cKO and wild-type mice. (B) Immunostaining with myosin7a (red) in apical, middle, and basal turns of cochleae from different groups. Scale bars = 20 μm. (C) Statistical analysis of HC number in (B). n = 3. Data are represented as means ± SD and analyzed by the unpaired two-tailed t-test.

**Supplementary figure 5.** The application of XMU-MP-1 has no effect on the HEI-OC1 damage caused by neomycin. (A) HEI-OC1 cells were treated with XMU-MP-1 for 24 h. Cell viability was then determined CCK8 assays. n = 5. Data are shown as means ± SD. (B) HEI-OC1 cells were treated with XMU-MP-1 and neomycin for 24 h. Cell viability was then determined CCK8 assays. n = 3. Data are shown as means ± SD. * represents the comparison with control group; ns, # represents the comparison with the neomycin group. (C) Cleaved-caspase 3 (red) and DAPI (blue) double staining showing apoptotic HEI-OC1 cells after neomycin-only and neomycin + XMU-MP-1 treatments. Scale bar = 50 μm. (D) Statistical analysis the proportion of caspase 3+ cells in (C). Data are shown as means ± SD. n = 6. Data were analyzed by the one-way ANOVA. ns = not significant, *p < 0.05, **p < 0.01, and ***p < 0.001.

**Supplementary figure 6.** AAV transfection efficiency. (A) Immunostaining with myosin7a (red) and HA (green) in cochleae of mice with and without AAV injection. Scale bars = 20 μm.

| Gene | 5’ to 3’ Forward Primer Sequence | 5’ to 3’ Reverse Primer Sequence |
| --- | --- | --- |
| Nf2 | CAGAAGCTCTTAGAAATCGCCA | GTGGTGGTGGAATTGGGTTCA |
| Sav1 | CTGTCCCGCAAGAAAACCAAA | AATGAAGGCATGAGATTCCGC |
| Wwc1 | TGCTGAGGGAAACCAAAGCC | CTGGACCATAGGTCGGAGTG |
| Lats1 | AAAGCCAGAAGGGTACAGACA | CCTCAGGGATTCTCGGATCTC |
| Yap1 | ACCCTCGTTTTGCCATGAAC | TGTGCTGGGATTGATATTCCGTA |
| Ankrrd1 | GCTGGTAACAGGCAAAAAGAAC | CCTCTCGCAGTTTCTCGCT |
| Birc5 | GAGGCTGGCTTCATCCACTG | CTTTTTGCTTGTTGTTGGTCTCC |
| Ccn2 | GGGCCTCTTCTGCGATTTC | ATCCAGGCAAGTGCATTGGTA |

**Table S1.** Primer sequences.
